# Myosins, Actin and Autophagy

**DOI:** 10.1111/tra.12410

**Published:** 2016-05-31

**Authors:** Antonina J. Kruppa, John Kendrick‐Jones, Folma Buss

**Affiliations:** ^1^ Cambridge Institute for Medical ResearchUniversity of Cambridge, Cambridge Biomedical CampusWellcome Trust/MRC Building, Hills RoadCambridgeCB2 0XYUK; ^2^ MRC Laboratory of Molecular BiologyFrancis Crick AvenueCambridge Biomedical CampusCambridgeCB2 0QHUK

**Keywords:** actin, autophagy, lysosome, myosin

## Abstract

Myosin motor proteins working together with the actin cytoskeleton drive a wide range of cellular processes. In this review, we focus on their roles in autophagy – the pathway the cell uses to ensure homeostasis by targeting pathogens, misfolded proteins and damaged organelles for degradation. The actin cytoskeleton regulated by a host of nucleating, anchoring and stabilizing proteins provides the filament network for the delivery of essential membrane vesicles from different cellular compartments to the autophagosome. Actin networks have also been implicated in structurally supporting the expanding phagophore, moving autophagosomes and enabling efficient fusion with the lysosome. Only a few myosins have so far been shown to play a role in autophagy. Non‐muscle myosin IIA functions in the early stages delivering membrane for the initial formation of the autophagosome, whereas myosin IC and myosin VI are involved in the final stages providing specific membranes for autophagosome maturation and its fusion with the lysosome.

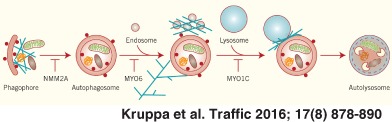

## Autophagy

Macroautophagy, hereafter referred to as autophagy, is a tightly regulated pathway that sequesters cytoplasmic material, damaged organelles, misfolded protein aggregates or invading pathogens into characteristic double‐membrane structures called autophagosomes, which fuse with lysosomes to allow content degradation 1. During nutrient starvation, this process of self‐eating is essential for cell survival. Thus, by turning over dysfunctional intracellular proteins and promoting the recycling of nutrients, autophagy plays a crucial role in maintaining cellular homeostasis 2. Furthermore, disruption of this pathway has been linked to a growing number of human diseases including cancer and neurodegeneration 3, 4, 5.

In this review, we will present a brief overview of the current knowledge of the autophagy pathway and highlight key role(s) of the actin cytoskeleton, myosin motors and associated cargo adaptor proteins in different stages of this vital cellular process.

## Actin in Autophagy

In eukaryotic cells, actin is one of the most abundant and highly conserved proteins. The 42‐kDa globular actin (G‐actin) monomer can polymerize into filamentous actin (F‐actin) 6. Actin filaments are polar double‐stranded polymers with a right‐handed helical twist: they have fast polymerizing barbed (+) ends and slow polymerizing pointed (−) ends. The assembly of actin filament networks is crucial for forming many subcellular structures and performing numerous cellular functions 7. Most aspects of actin dynamics are regulated by the Rho family of GTPases; the most important members are RHOA, CDC42 and RAC1, which play pivotal roles in shaping the actin cytoskeleton and involve numerous downstream effector proteins 8. Spontaneous actin filament assembly, however, is thermodynamically unfavourable and is limited by the nucleation of actin dimers and trimers 9. Therefore, cells use factors that nucleate actin to overcome this rate‐limiting step of *de novo* actin filament formation, such as the ARP2/3 complex, formins and nucleation promoting factors 10. The 220‐kDa ARP2/3 complex comprises seven highly conserved proteins (ARP2, ARP3 and ARPC1–ARPC5) that can nucleate both filaments and branched networks. It binds to a pre‐existing actin filament and initiates the assembly of a new filament at a ∼70° angle, which elongates at its barbed end and is capped by ARP2/3 at its pointed end 11. Mammalian cells also express several nucleation promoting factors containing diverse amino‐terminal sequences that enable different modes of actin regulation and functions 12, including WHAMM (WASP homolog associated with actin, membranes and microtubules) and WASH (WASP and SCAR homology) 10. Actin association with these different regulatory proteins leads to filament polymerization and a wide variety of cellular architectures.

The general importance of the actin cytoskeleton in autophagy was demonstrated using F‐actin depolymerizing drugs, such as cytochalasin D and Latrunculin B, which inhibit autophagosome formation 13, 14. In addition, deletion of core elements of the autophagy machinery also impacts on actin filament assembly, since F‐actin is disassembled in ATG7 knockout mouse embryonic fibroblasts (MEFs) during starvation‐induced autophagy 15. In this review, we will summarize the evidence that demonstrates the importance for dynamic actin reorganisation during different stages of the autophagy pathway.

## Myosins in Autophagy

In the human genome, 39 myosins belonging to 12 distinct classes are expressed 16. One of the basic cellular functions of the non‐muscle myosins is the sorting, transport and distribution of vesicles, protein complexes, membranes and other specific cargoes along the dynamic actin cytoskeleton to maintain the health and integrity of the cell. So far three myosins have been shown to play essential roles in specific steps of the autophagy pathway: non‐muscle myosin IIA (NMM2A) 17, myosin IC (MYO1C) 18 and myosin VI (MYO6) 19, 20. NMM2A operates in the early stages of autophagy during the initiation and expansion of the phagophore, whereas MYO6 and MYO1C are involved in the late stages of autophagosome maturation and fusion with the lysosome, respectively. Given the importance of actin filament dynamics during autophagy and the many roles of myosin motors in regulating actin filament organization as well as in moving cargo along actin filament tracks, it is very likely that a number of other myosins are also involved in specific stages of this pathway.

## Autophagy is a Multi‐Step Process

**Figure 1:**
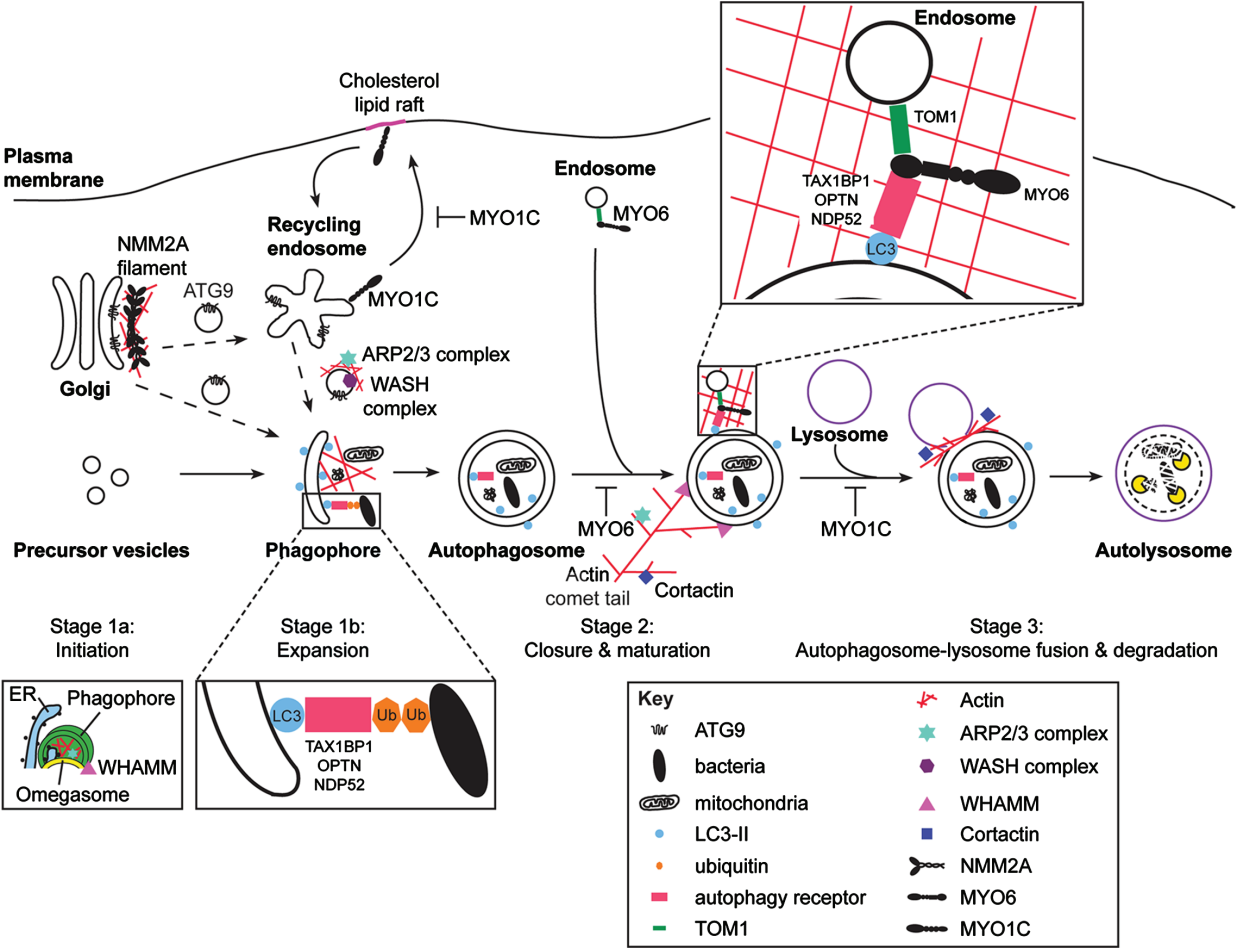
**The role of actin and myosins in mammalian autophagy.** This schematic diagram depicts the stages at which myosin motors and the actin cytoskeleton are thought to act in the autophagy pathway. Stage 1a (Initiation): Under conditions of low nutrients or stress, which is sensed by mTORC1 or AMPK, the ULK complex initiates autophagosome biogenesis. The ULK complex targets a class III PI3K complex that produces PI3P on omegasomes. Stage 1b (Expansion): NMM2A mini‐filaments and the actin cytoskeleton regulated by the WASH complex are important for the formation of ATG9 vesicles that cycle between the TGN and endosomes, and together with several other membrane sources contribute to expansion of the phagophore. The actin nucleator, ARP2/3, and nucleation promoting factors, such as WHAMM, induce a scaffold of branched actin networks inside the growing phagophore dome. The MYO6 adaptors and autophagy receptors – TAX1BP1, NDP52 and OPTN – may form the bridge between ubiquitinated bacteria or mitochondria and LC3 on the phagophore membrane. Stage 2 (Closure and maturation): LC3 is required for elongation of the phagophore and the closed autophagosome undergoes further maturation by fusion with endosomal compartments delivered by MYO6, which binds to endosomes via its adaptor protein, TOM1. MYO6 and associated endosomal cargo may be recruited to autophagosomal membranes by binding to autophagy receptors. WHAMM‐dependent actin comet tail formation and stabilization by cortactin leads to movement of autophagosomes. Stage 3 (Autophagosome‐lysosome fusion and degradation): Autophagosomes fuse with lysosomes and the content is degraded by hydrolytic enzymes. Autolysosome formation requires the correct lipid composition of the individual organelles, which involves MYO1C‐dependent cholesterol‐enriched lipid raft trafficking, and a cortactin‐dependent remodelling of the F‐actin network.

This dynamic pathway from autophagosome biogenesis to lysosomal degradation can be divided into several morphological and functional stages: (i) initiation and phagophore expansion, (ii) autophagosome closure and maturation, and (iii) autolysosome formation and degradation (Figure 1). Autophagy is upregulated when cells are under stress and nutrient starvation is the best understood form of autophagy induction.

### Stage 1: initiation and phagophore expansion

Under conditions of low nutrients or other stress signals, autophagosome biogenesis is triggered by the UNC51‐like kinase (ULK; ATG1 in yeast) complex (ULK1 or 2, ATG13, ATG101 and FIP200), which is regulated by the cellular energy sensor, AMP‐activated protein kinase (AMPK), and the nutrient sensor, mTOR complex 1 (mTORC1) 21. The activated ULK complex targets a class III PI3K complex consisting of VPS34, beclin 1, VPS15 and ATG14L that locally produces phosphatidyl‐inositol 3‐phosphate (PI3P) on membrane structures called omegasomes 22, 23. These cup‐shaped (omega‐shaped) extensions of the endoplasmic reticulum (ER), which are enriched in the PI3P‐binding, double FYVE domain‐containing protein (DFCP1) 24, mark the initial site of autophagosome formation. The omegasome forms a ring‐like base and physically demarcates the outer edges of the protruding, highly curved dome‐like phagophore that appears to be sandwiched between two sheets of ER, a so‐called ER ‘cradle’ 25, 26.

In addition to the ER, other membrane sources also contribute to the propagation and elongation of the phagophore membrane, including the plasma membrane 27, endosomes 28, 29, 30, mitochondria 31 and the Golgi complex. For example, vesicles containing the multi‐pass transmembrane protein ATG9 are trafficked from the *trans*‐Golgi network (TGN) to the phagophore where they are thought to deliver membranes for expansion 32, 33, 34.

### Stage 2: autophagosome closure and maturation

Two ubiquitin‐like conjugation systems involving numerous autophagy (ATG) proteins ultimately lead to the covalent modification of LC3 (microtubule‐associated protein 1 light chain 3) with phosphatidylethanolamine (PE) 35, 36, 37. LC3 is required for the elongation and remains associated with the completed autophagosome, while omegasomes are retracted back into the ER. Autophagosomes then fuse with endosomal compartments (early endosomes or multivesicular bodies, MVBs) to form amphisomes 38, 39.

### Stage 3: autolysosome formation and degradation

Amphisomes or autophagosomes fuse with lysosomes giving rise to the autolysosome 40. In this acidic environment, lysosomal hydrolases degrade the contents and recycle these metabolites including amino acids, fatty acids, glucose and iron back to the cytosol 2.

## Selective Autophagy

Although autophagy has long been considered a non‐selective degradative pathway for cytoplasmic material during nutrient deprivation, more recently cargo‐specific autophagy receptors have been identified that lead to the selective degradation of specific cargoes, such as damaged mitochondria, protein aggregates and invading pathogens 41, 42. These autophagy receptors, including p62/SQSTM1 and its paralogue NBR1, TAX1BP1 and its paralogue NDP52, and optineurin (OPTN), confer substrate specificity by selectively binding to their cargo. They recognize and capture their cargoes marked for degradation with a ubiquitin tag via their ubiquitin‐binding domains (UBDs) or via ubiquitin‐independent means 43 and simultaneously bind to LC3 on autophagosomal membranes via their LC3 interacting region (LIR) (Figure 1) 44. Three of the known autophagy receptors – OPTN, NDP52 and TAX1BP1 – are also MYO6 adaptor proteins implying actin‐based transport is important in autophagy. Intriguingly, the MYO6 and ubiquitin binding sites overlap in the C‐terminal zinc finger domains of NDP52 and TAX1BP1 20, 45, and in the C‐terminal coiled coil region of OPTN 46 suggesting that ubiquitin and MYO6 binding are mutually exclusive 47. Interestingly, the autophagy receptors that bind MYO6 have been shown to play partially redundant functions in selective autophagy of the evolutionary related substrates, bacteria and mitochondria, which are targeted by xenophagy and mitophagy, respectively 48.

### Xenophagy mediates antibacterial defence

Pathogens including the *Salmonella* bacterium invade cells and become resident in a *Salmonella*‐containing vacuole (SCV), where they proliferate. The first danger signal marking the escape of bacteria from the vacuole into the cytosol is the rupture of these vacuolar membranes 49. This leads to the exposure of host galactose‐containing glycans on damaged SCVs that are recognized by cytosolic lectins of the Galectin family, which can trigger recruitment of xenophagy receptors. For example, Galectin‐8 is the danger signal on the damaged SCV that induces ubiquitin‐independent xenophagy through recruitment of NDP52 49, 50. After entering the cytosol, the escaped bacteria become rapidly decorated with a variety of ubiquitin chains, which then directly recruit the autophagy receptors, including NDP52, TAX1BP1 and OPTN, to the bacterial surface and target it for clearance by xenophagy 20.

### Mitophagy clears dysfunctional mitochondria

To maintain mitochondrial homeostasis, damaged mitochondria are targeted to autophagosomes for degradation using the PINK1/Parkin‐dependent pathway 51. A hallmark of mitochondrial damage is the loss of membrane potential, which leads to stabilization of the kinase PINK1 on the outer mitochondrial membrane 52, where it phosphorylates ubiquitin. Several groups have shown that this recruits and activates the E3 ubiquitin ligase, Parkin 53, 54, 55, 56, 57, which is further activated by PINK1‐dependent phosphorylation of a homologous site in its ubiquitin‐like (UBL) domain as a positive feedback mechanism 58, 59. Parkin then ubiquitinates outer mitochondrial membrane proteins, thereby amplifying the mitophagy signal 60, 61, 62, 63. Parkin is also involved in the formation of the ubiquitin coat surrounding *Mycobacterium tuberculosis* 64, further highlighting the functional similarities between mitophagy and xenophagy.

Genetic knock out of five autophagy receptors (OPTN, NDP52, TAX1BP1, p62 and NBR1) using the CRISPR/Cas9 system leads to a block in mitophagy 63. TAX1BP1, OPTN and NDP52 are functionally redundant autophagy receptors and the latter two require Tank‐binding kinase 1 (TBK1)‐dependent phosphorylation to enhance their ability to bind ubiquitin chains 65, 66, whereas NBR1 and p62 are dispensable for mitophagy 63, 67. The ubiquitin kinase activity of PINK1 induces mitophagy by recruiting the autophagy receptors to dysfunctional mitochondria 63. These, in turn, have been suggested to recruit the early autophagic machinery required for initiation (ULK complex) and PI3P‐binding (DFCP1) by a yet unknown mechanism, and are known to bind LC3 for phagophore expansion as described in the previous section 63, 67.

## The Roles of Actin and Myosins in Autophagy

### Stage 1: initiation and phagophore expansion

#### Structural role of actin in phagophore biogenesis

Actin filaments have been shown to colocalize with early omegasomes before their association with LC3 suggesting a role for actin in the initial stages of autophagosome formation linked to the PI3P generation step (stage 1a, Figure 1; 14). Upon autophagy induction, the actin nucleator, WHAMM, moves to omegasomes by an unknown mechanism and recruits the ARP2/3 complex 68. In response to starvation, branched actin networks containing the ARP2/3 complex polymerize inside the expanding phagophore (stage 1b, Figure 1; 69), which may provide the cytoskeletal framework for generating membrane curvature as the phagophore expands so that the ends can ultimately fuse to form the autophagosome 70. Pharmacological inhibition or depletion of CapZ (*CAPZB*), an actin capping protein that binds to the barbed end of actin filaments 71, abolishes the formation of branched actin networks. This leads to the collapse of the dome‐shaped phagophore resulting in elongated tubular structures (positive for both DFCP1 and LC3). These observations suggest that actin filament networks may have a structural scaffolding role in generating the shape of the phagophore 69, 70. In summary, branched actin networks are required for omegasome maintenance, phagophore expansion and autophagosomal membrane shaping as they provide a three‐dimensional structure for support and assembly of the omegasome and phagophore.

#### Non‐muscle myosin IIA operates during phagophore initiation and expansion

NMM2 is ubiquitously expressed in all eukaryotic cells 16. This myosin, like muscle myosin II, is composed of two 230 kDa heavy chains that form two globular motor domains and dimerise into a coiled‐coil tail domain. Two 20 kDa regulatory light chains (RLCs) and two 17 kDa essential light chains (ELC) bind to and stabilize the lever arm between the motor and tail domain 72. The motor domain contains the ATP and actin binding sites, while the lever arm amplifies the conformational changes derived from the actin‐activated ATP hydrolysis into mechanical movement of the motor domain along actin filaments. The tails of non‐muscle myosin II assemble side‐by‐side into short bipolar filaments composed of ∼24–28 molecules, which interact with actin filaments to form the ‘contractile unit’ 73.

Three different non‐muscle myosin II isoforms (NMM2A, 2B and 2C) are expressed in mammalian cells. NMM2A is encoded by myosin heavy 9 (MYH9), 2B by MYH10 and 2C by MYH14 74. Although all these isoforms have slow actin‐activated ATPase activities, their kinetic properties differ: non‐muscle myosin IIA has the highest rate of ATP hydrolysis and moves the fastest along actin filaments, whereas non‐muscle myosin IIB has the highest duty ratio (the proportion of the ATPase cycle spent bound to actin) and a high binding affinity for ADP 75. NMM2A is involved in dynamic events, such as translocation and active cell migration 76, whereas NMM2B is required for tension maintenance and structural cell rigidity 77. Thus, the kinetic properties of these isoforms appear to govern their cellular roles 78. The wide spectrum of functions and the distinct contributions of each of these isoforms in development, cell migration, adhesion, polarity and disease have been reviewed in detail elsewhere 79.

The activity of the NMM2s is regulated by RLC phosphorylation by more than 12 different kinases 80, 81, 82. At least *in vitro* non‐phosphorylated NMM2 exists in a compact inactive state (10S state) in which the folded tail interacts with the motor domains. This inhibits its ATPase activity by reducing its rate‐limiting Pi release rate ∼1000 fold and prevents filament assembly 83, 84, 85. RLC phosphorylation releases the tail and unfolding allows filament assembly and ATPase activation.

During autophagosome biogenesis, NMM2A is activated downstream of the serine/threonine kinase ATG1 (mammalian ULK1) that is essential for induction of autophagosome formation under starvation conditions (stage 1a, Figure 1) 86. NMM2A is known to be recruited to membranes at the TGN possibly via its tail domain interaction with Rab6 87 and is thus thought to be involved in the formation of transport vesicles at the Golgi complex. The ULK complex has been shown to regulate the delivery of ATG9‐containing vesicles from the TGN through endosomes to the expanding phagophore (stage 1b, Figure 1), thereby providing the membrane essential for biogenesis 88. In *Drosophila* and in mammalian cells, the ULK1/ATG1 kinase phosphorylates and activates a novel myosin light chain kinase (Spaghetti‐squash activator, Sqa, in *Drosophila* and zipper‐interacting protein kinase, ZIPK, in mammalian cells), which in turn phosphorylates the myosin RLCs during starvation and leads to activation of NMM2A 17. The phosphorylated NMM2A then assembles into bipolar filaments, which together with actin may form an actomyosin filament network that could provide the force or tension required for ATG9 transport vesicle formation at the TGN or could serve as tracks to deliver the ATG9 membranes from the Golgi complex to the sites of phagophore expansion in the cell. Additional myosin motors may be required to actually translocate the membrane vesicles along actin filaments, as activated NMM2A, when assembled into myosin mini‐filaments, is unable to bind cargo via its C‐terminal tail domain and thus function as a transporter.

#### Actin‐dependent ATG9A trafficking for phagophore expansion

The trafficking of ATG9A through early and recycling endosomes to the site of autophagosome formation (stage 1b, Figure 1) requires the presence of actin binding proteins, such as Annexin A2, and actin nucleators, including ARP2/3, Spire1 and the WASH complex 10, 89, 90. These actin‐associated proteins have been proposed to modulate actin assembly on endosomes. Depletion of Annexin A2 results in ATG9A accumulating in early endosomes, reduced trafficking of ATG9A to recycling endosomes, and failure of ATG9A vesicles to reach the phagophore 89. Knockdown of the WASH complex increases colocalization of ATG9A with the TGN and impairs autophagosome formation 90. In short, localized generation of F‐actin patches on ATG9A vesicles by actin nucleating factors is crucial for the correct trafficking of ATG9A vesicles from endosomes to autophagosomes where they are thought to deliver membrane for phagophore expansion 32, 33.

Interestingly, inhibition of the Rho‐associated kinase (ROCK1), which is a downstream effector of Rho GTPases, causes accumulation of enlarged autophagosomes 91. Phagophore extension leading to these large autophagosomes is not driven by ATG9A vesicles as their trafficking is not affected by ROCK inhibition. Another study, however, showed that inhibition of RHOA or ROCK activity reduced the number of autophagosomes in cells under starvation conditions. In contrast, loss of RAC1 activity upregulates starvation‐induced autophagy, whereas changes in CDC42 activity have no impact on the pathway 14.

### Stage 2: autophagosome closure and maturation

#### Actin comet tails on maturing autophagosomes

Actin comet tails, which are branched actin filaments produced by the ARP2/3 complex on the surface of particles, have been implicated in generating the force that drives the movement of bacterial pathogens, endosomes and lysosomes 92, 93. The ARP2/3 complex activator, WHAMM, was recently demonstrated to have a role in actin‐comet tail formation on autophagosomes (stage 2, Figure 1), which also associates with the branch stabilizing protein, cortactin 68. Interfering with actin polymerization by pharmacologically inhibiting the ARP2/3 complex, by knocking down WHAMM or by blocking the interaction of WHAMM with ARP2/3 using mutagenesis, inhibited comet tail formation and reduced the size and number of autophagosomes. It was proposed that the forces generated by actin polymerization are harnessed to move autophagosomes during starvation‐induced autophagy 68, 94.

#### Myosin VI and its cargo adaptors link endocytosis to autophagosome maturation

Myosin VI (MYO6) is the only myosin that moves towards the minus end (pointed end) of actin filaments, in the opposite direction to all the other myosins 95. This unique myosin plays essential roles in membrane trafficking pathways 96, 97, 98 and has been shown to mediate the delivery of endosomal membranes to autophagosomes, a process required during autophagosome maturation 19, 47.

MYO6 has unique structural and functional properties. It consists of a conserved N‐terminal motor domain, a short lever arm domain binding to one calmodulin, a tail domain with a three‐helix bundle, a single alpha helical domain (SAH) and a C‐terminal cargo‐binding domain that interacts with a host of specific adaptor proteins. There is a unique 53‐amino‐acid insert – the reverse gear – in between the motor domain and the lever arm that is stabilized by binding a calmodulin and is responsible for the reverse directionality of MYO6 movement along actin filaments by repositioning of the lever arm 99, 100.

MYO6 displays a number of unusual features, not only the reverse directionality, but also a large step size of about 36 nm despite a short conventional lever arm. To account for this large step size, the proximal three‐helix bundle in the tail may unfold to form a lever arm extension 101, 102. In contrast to NMM2 (a dimer) or MYO1C (a monomer), MYO6 has been suggested to undergo monomer‐dimer transitions regulated by cargo adaptor proteins 103. This allows greater functional diversity for MYO6 in cells, when switching between a monomer and a dimer state dependent on the cellular context. As a monomer, MYO6 could perform load‐dependent anchoring functions and as a dimer, it could move processively and transport cargo over short distances along actin filaments. Whether both these functional states of MYO6 occur *in vivo* still requires further investigation.

Since MYO6 is the only pointed‐end directed myosin motor, it is involved in a unique spectrum of cellular functions ranging from anchoring the actin cytoskeleton to the plasma membrane in the highly specialized stereocilia of the inner ear, to clathrin‐mediated endocytosis in polarized epithelium, to exocytosis/secretion at the Golgi and plasma membrane, to the trafficking and sorting of cargo at early endosomes 104. These diverse cellular functions of MYO6 are mediated by cargo adaptor proteins, which bind to specific sites in the C‐terminal cargo‐binding domain via either an RRL motif (adaptor proteins NDP52, OPTN, TAX1BP1 and GIPC) or a WWY motif (TOM1, LMTK2 and DAB2) 45, 98. In addition, the tail can bind to ubiquitin and contains a phospholipid‐binding domain 98, 105. There is now mounting evidence that these adaptor proteins not only bind to select cargo and mediate their delivery to specific cellular compartments, but they also fine‐tune the activity of the MYO6 motor and regulate its monomer‐dimer state 103, 106.

A number of MYO6 adaptor proteins – NDP52, OPTN, TAX1BP1 and TOM1 – have important roles in both the early and late phases of autophagy (Figure 1). NDP52, TAX1BP1 and OPTN function as autophagy receptors to initially target invading pathogens and damaged organelles, such as mitochondria, for selective autophagy 107 (as described earlier). However, so far there is no evidence that MYO6 is involved in these early phases of autophagy. Depletion of MYO6 leads to an accumulation of autophagosomes in nutrient‐starved cells suggesting a breakdown in the final stages of autophagosome maturation and fusion with the lysosome 19. Thus, OPTN, NDP52 and TAX1BP1 may have multiple roles in autophagy: initially acting as receptors for cargo recognition and autophagosome biogenesis and then in later stages, by binding directly to MYO6, they may be involved in autophagosome maturation and fusion with the lysosome.

MYO6 is known to function at distinct steps in endocytosis 96, 104 and has been shown to bind via its WWY motif to the cargo adaptor TOM1 (an ESCRT protein component) in the early endosomal compartment just beneath the plasma membrane, where it may be involved in the sorting and delivery of endosomal membrane vesicles and components to the late endosome (or multivesicular bodies, MVB) and the autophagosome. How the ESCRT‐TOM1 complex works together with MYO6 to deliver these membrane vesicles and how their subsequent fusion with the autophagosome occurs is not yet known. However, we speculate that MYO6 by binding via its WWY motif to the endosomal cargo adaptor TOM1 may sort and bring the required endosomal membrane vesicles into close contact with the autophagosome by tethering them to the surrounding actin filament network and then aligning them by binding via its RRL motif to TAX1BP1, NDP52 or OPTN on the outer surface of the autophagosome 19. In this way, the membrane vesicles are positioned to allow for SNARE‐mediated membrane incorporation into the autophagosome and subsequent fusion with the lysosome. The dual motor properties of MYO6 as a potential processive dimeric motor involved in short range transport or as a monomeric tether regulated through different adaptor proteins are consistent with this model 47. This role for MYO6 in autophagosome maturation and degradation is not only required during starvation‐induced autophagy 19, but also for selective autophagy pathways. In xenophagy, MYO6 is recruited to ubiquitinated *Salmonella* and loss of MYO6 results in a hyper‐proliferation of ubiquitin‐positive *Salmonella* in autophagosomes 19, 20. However, it should be stressed that given MYO6's involvement in diverse membrane trafficking pathways 96, 104, it could also have autophagy receptor‐independent functions in autophagosome maturation.

### Stage 3: autolysosome formation and degradation

#### F‐actin network in autophagosome‐lysosome fusion

The actin cytoskeleton is also required for the fusion of autophagosomes with lysosomes (stage 3, Figure 1) during selective autophagy, but in this study is dispensable for starvation‐induced autophagy 108. The actin remodelling factor, cortactin, is recruited to autophagosomes by the ubiquitin‐binding deacetylase, HDAC6. Cortactin induces actin polymerization and rearrangement of actin filaments by recruiting the ARP2/3 complex, which stimulates the local assembly of an F‐actin network to enable the efficient fusion of autophagosomes and lysosomes for subsequent degradation of protein aggregates 108. Depletion of cortactin leads to loss of F‐actin network formation accompanied by a defect in protein aggregate clearance due to a block in autophagosome‐lysosome fusion. Further evidence that actin plays a role in autophagosome‐lyososome fusion comes from *in vitro* assays in which Latrunculin A treatment reduced fusion of purified autophagosomes with lysosomes, which could be restored by adding back purified actin 108.

#### Myosin IC links cholesterol trafficking and autophagosome‐lysosome fusion

Myosin IC (MYO1C) is a monomeric myosin of class I, which associates with cholesterol‐enriched lipid rafts and regulates cellular cholesterol homeostasis 109. MYO1C has a single motor domain, which is followed by a neck region with three calmodulin‐binding IQ motifs and a short tail containing a myosin I tail homology domain (TH1). This TH1 domain contains a positively charged lipid‐binding region and a pleckstrin homology (PH) domain that binds specifically to phosphatidylinositol 4,5 bisphosphate (PIP2) and is required for recruitment of MYO1C to lipid membranes 110, 111. The structural and kinetic characteristics of MYO1C determine its cellular behaviour and range of activities 112. MYO1C is a slow motor overall as its activity is limited by the slow release of phosphate during its ATPase cycle. However, it is a ‘strong’ motor when compared with the other myosins of class I; it can translocate heavy cargo and only stalls at forces of about 2.5 pN 113. This implies that MYO1C has been designed to actually translocate large loads at a slow pace, whereas other class I myosins are much more sensitive to load and therefore are better adapted to tether heavy cargo and, e.g., maintain tension at the membrane/cytoskeleton interface 114.

MYO1C is widely expressed in most eukaryotic cells. It is enriched in dynamic regions of the plasma membrane, but is also present in the nucleus and on intracellular tubules and vesicles 115. Its activity is required for a wide range of critical functions in a variety of cell types and is often linked to dynamic reorganisation of actin structures underneath the plasma membrane as seen in membrane ruffles, lamellipodia and filopodia 116, 117. Moreover, in the exocytic/recycling pathway, MYO1C mediates the delivery of a variety of cargoes to the plasma membrane 118.

An insight into the molecular role of MYO1C in these diverse pathways was provided by the discovery of a spatial association between MYO1C and lipid rafts. This motor is recruited through its PH‐domain to cholesterol‐enriched micro‐domains at the plasma membrane and also to membrane tubules in the endocytic recycling pathway 109. Reducing the expression of functional MYO1C has a dramatic impact on intracellular cholesterol trafficking, which leads to reduced levels of lipid‐raft associated proteins at the plasma membrane and accumulation of these proteins in the perinuclear recycling compartment. This phenotype suggests a role for MYO1C in lipid raft recycling either by regulating sorting of cargo into lipid rafts at the recycling compartment or by promoting tubule formation at the recycling endosome or by mediating fusion of exocytic carriers at the plasma membrane 109.

The loss of MYO1C not only changes the cellular distribution of lipid rafts, but also leads to a significant increase in total cellular cholesterol and enlargement of late endosomes and lysosomes. This swelling of the late endocytic compartment in MYO1C‐depleted cells is, however, not caused by storing the increased cholesterol in lysosomes as described for lysosomal storage diseases, such as Niemann‐Pick Type C 119. These MYO1C‐dependent changes in lysosome morphology do not affect the overall function of these lysosomes and their proteolytic capacity, since degradation of endocytic cargo and receptors is unchanged. However, depletion of MYO1C leads to a dramatic accumulation of autophagocytic organelles, indicating a selective block in autophagosome‐lysosome (stage 3, Figure 1) or amphisome‐lysosome fusion and hence a defect in the degradation of these autophagocytic structures. It is well‐established that increases in the overall cellular lipid load together with changes in the lipid composition of autophagosomes and lysosomes affects their ability to fuse with each other 120. Therefore, the established function of MYO1C in regulating the intracellular distribution of cholesterol‐enriched membranes, which has been shown to elevate cellular cholesterol levels, may underlie the mechanism by which MYO1C modulates autophagy. Increased lipid levels may impact on autophagosome clearance, but changes in lipid trafficking may also specifically alter the lipid and cholesterol content of autophagosomes and lysosomes, which reduces their ability to fuse. Thus, the current evidence suggests that the function of MYO1C in lipid raft recycling changes the overall cholesterol distribution, which has an indirect impact on autophagosome‐lysosome fusion by changing the lipid composition of these organelles.

## Summary

In this review, we have summarized how actin networks regulated by an array of associated nucleating, polymerizing and stabilizing proteins generate membrane curvature and act as a structural scaffold for the expanding phagophore (stage 1, Figure 1), move autophagosomes by forming actin comet tails (stage 2, Figure 1), and enable efficient fusion of autophagosomes with lysosomes (stage 3, Figure 1). While MYO6 is the only myosin directly associated with autophagosomal compartments via its cargo adaptor proteins/autophagy receptors during the maturation stage (stage 2, Figure 1), NMM2A and MYO1C impact on the autophagy pathway indirectly through their well‐defined roles in ‘general’ membrane trafficking. NMM2A together with actin regulatory proteins ensure formation of ATG9 vesicles at the TGN and delivery of essential membrane from the recycling endosome to the site of phagophore expansion (stage 1b, Figure 1). In contrast, MYO1C's important function in trafficking cholesterol‐enriched membranes leads to a block in autophagosome‐lysosome fusion (stage 3, Figure 1). It is likely that many more myosins moving along and tethering to dynamic actin filament networks are involved especially in the early and late stages of autophagy.

Dysfunction of this pathway occurs in a growing number of human diseases that either affect multiple systems, e.g., cancer, defects in the immune response, metabolic dysfunction and vascular disease or target specific organs, such as the central nervous system, the heart, skeletal muscle or bones 4. The importance of mutations and/or deletions in myosins in human diseases is an emerging area of research, however, at present, it is too early to predict whether these human pathologies are caused by loss of myosin or actin function in the autophagy pathway. Changes in NMM2A expression have been linked to cancer and to multiple systems including macrothombocytopenia, cataracts, deafness and glomerulosclerosis 79. Mutations in MYO6 and MYO1C are a major cause of deafness in humans, however, so far a detailed phenotypic analysis of the affected patient families for other pathologies related to dysfunctional autophagy have not yet been performed.

## Supporting information

Editorial Process
