# Supplementary material for: Myosins, Actin and Autophagy
Source: Traffic. 2016 May 31;17(8):878–90. doi: 10.1111/tra.12410 (PMC4957615; doi:10.1111/tra.12410)
Supplement: Supplementary file 1 — Editorial Process [file TRA-17-878-s001.docx]

| Title: | **Myosins, actin and autophagy** |
| --- | --- |
| Authors: | Antonina J. Kruppa, John Kendrick-Jones, and Folma Buss |
| Article Type: | Review |
|  |  |
| Monitoring Editor | Sharon Tooze |
| Date Submitted | 24 March 2016 |
| Date for Decision 1 | 17 April 2016 |
| Date Resubmitted | 27 April 2016 |
| Accepted | 2 May 2016 |

**Decision and Reviews**

Dear Dr. Buss,

Thank you for taking the time recently to write your review “Myosins, actin and autophagy” for Traffic. I asked two colleagues who are experts in the field to review this paper and their verbatim comments are appended below. I agree with the referees that this is a well-written and timely review that will be of significant interest to the readers of Traffic, in particular as it is one of the first comprehensive reviews of the topic. The referees have made a number of suggestions to extend and correct the discussion and to include additional references that I agree will improve this review. In particular, I ask that you consider reorganizing the review as described by referee 2, but this is not a requirement for acceptance, and I would place more priority on doing this rather than discussing actin regulators.

Although I cannot accept your manuscript for publication at this point, I believe that you will be able to address the referees concerns and I look forward to receiving your revised manuscript. To expedite handling when you resubmit please be sure to include a response outlining how you have addressed each of the referees’ concerns.

Sincerely

Sharon A. Tooze, Ph.D.
Co-Editor
________________________________________________________

Referee's Comments to the Authors


Referee: 1

Comments to the Author
Actin cytoskeleton plays a pivotal role in a variety of cellular processes. The myosin superfamily proteins are actin-based motors consisting of more than 100 members. Recent studies have indicated the involvement of actin and myosin in autophagy. Here, authors discussed recent progresses on the role of actin and myosin in the regulation of autophagy pathway. The article covers latest studies in the field and is logically organized.

The area that authors haven’t addressed much is the role of actin regulators in autophagy. It is known that Rho family GTPases are essential for actin cytoskeleton organization and dynamics. Recently, several reports have implicated a role of Rho GTPases in autophagy. The authors should include these findings in their article.

Dysregulation of autophagy has been associated with various human diseases. The authors should elaborate more on the role of actin and myosin in autophagy-related diseases.


Referee: 2

Comments to the Author
In this manuscript, the authors review the current literature on the interconnections between actin, myosins and autophagy. The topic is timely as we are now starting to understand the role of actin and myosins in autophagy at a more detailed and mechanistic level. Moreover, the topic has not recently been extensively reviewed elsewhere.
The authors start by giving an overview of the different stages of the autophagy pathway and the different forms of selective autophagy. Then, they give an introduction to actin before they describe what is known about the role of actin in the different stages of autophagy. Finally they discuss the role of various myosins in autophagy.
General comment:
The manuscript is generally well written and covers well the current literature implicating actin/myosins in autophagy. However, it would have read better if the authors would better integrate the autophagy part (the first 4 pages) with the actin/myosin part. As it is written now, the reader does not encounter the main topic of the review (role of actin/myosin in autophagy) until half way down page 5. Moreover, when coming to the main part (page 5-10) the authors should aim to include more details about autophagy when they describe the studies implicating actin/myosin in autophagy, e.g. what kind of autophagy was studied (non-selective or selective autophagy), how autophagy was induced (starvation, rapamycin, …), how autophagy was inhibited (siRNA against e.g. ATG7, Bafilomycin 1, …) and which species used for the studies. Now, they often state that autophagy is affected without really mentioning the specific details. This is sometimes problematic as it can be misleading -- e.g. on page 6 when they discuss ref 65 (Lee et al; cortactin-HDAC6) they do not mention that this study found that HDAC6 and F-actin assembly were completely dispensable for starvation-induced autophagy.
The authors should also aim to better describe any controversial findings, e.g. in ref 54 (Aguilera et al) it was found that actin functions at a very early stage of autophagosome formation, before association of LC3, whereas Mi et al (ref 57) LC3 is seen together with the actin puncta.
It is also a problem that the authors sometimes draw conclusions by combining results from two (or more) independent papers (and species). One such example is at page 7 (left panel) where the authors write “By activating NMM2A, the ULK complex regulates the delivery of ATG9-containing vesicles from the TGN to the expanding phagophore (stage 2, Figure 1), thereby providing the membrane essential for biogenesis (86).” Ref 86 does not say anything about NMM2A!

Minor comments:
Page 2: add a couple references to the Autophagy introduction
Page 3, stage 2: they should add references to endosomes as sources of autophagy membrane (Longatti et al, JCB 2012; Knævelsrud et al, JCB 2013; Puri et al, Cell 2013)
Page 3, stage 3: when citing LC3 conjugation to PE they should include the original paper; Kabeya et al, EMBO J, 2000 (ref 16 here) in addition to refs 14-15. It is not shown in ref 16 that LC3 is required for autophagosome closure and this is also highly debated in the field. They should also include a reference to amphisomes.
Page 3, stage 4: they should also mention that degradation products can be recycled to the cytosol and reused.
The authors should be consistent in their citations, sometimes they write “as recently reviewed …” when they cite reviews, other times they cite a review without mentioning that this is a review.

Page 3; they should mention that not all forms of selective autophagy are ubiquitin-dependent.

Page 4, second chapter; “In contrast, TAX1BP1 shows no preference and binds equally to LC3B and LC3C (22), which allows recruitment of a wider variety of membranes containing different LC3 isoforms and may suggest less specialised roles of TAX1BP1 in bacterial autophagy.” We do not know if the different LC3 (and GABARAP) isoforms are found in different membranes, so it is difficult to conclude about specificity.

Page 4: “…require TBK1-dependent enhancement of their ability to bind poly-ubiquitin,…” This should be explained better.

Page 6, first paragraph: the authors write “Additional myosin motors may be required to actually translocate the membrane vesicles along actin filaments, as activated NMM2A, when assembled into myosin minifilaments, is unlikely to function as a cargo transporter.” Why not?

Page 8, right panel: The authors should use be consistent in their use of the term autophagy receptor. Sometimes they use adaptor proteins instead, e.g. “MYO6 has been suggested to undergo monomer-dimer transitions regulated by cargo adaptor proteins (95).” This mix-up is confusing in the figure, where a red bar is called autophagy receptor in the key inset, while adaptor is used in the figure.

Page 9, first paragraph: “Thus, OPTN, NDP52 and TAX1BP1 may have multiple roles in autophagy:
initially acting as receptors for cargo recognition and autophagosome biogenesis and then in later stages, by binding directly to MYO6, they may be involved in autophagosome maturation and fusion with the lysosome.” MYO6 could have autophagy-receptor independent functions in autophagosome maturation!

The figure: is nice, but maybe a bit crowded. The box showing actin within the omegasomes should be moved to under stage 1: initiation.

The following references are not complete in the reference list and should be checked: 22,26, 32, 33, 46, 60, 68,.

Ref. 64; the original paper should be cited (Kast et al, Curr Biol 2015) instead of the Autophagy puncta.

**Author Rebuttal**

Dear Sharon:

Thank you for your very positive letter and the thorough review of our manuscript “Myosins, actin and autophagy” by Antonina Kruppa, John Kendrick-Jones and Folma Buss. Overall we found the reviewers’ comments very helpful and constructive and the manuscript has been amended accordingly and is thus significantly improved. The manuscript has been amended according to the reviewers’ suggestions as follows:

Referee 1
1. “Recently, several reports have implicated a role of Rho GTPases in autophagy. The authors should include these findings in their article.
We have now included sections on RHOA, RAC, CDC42 and some of their downstream effector proteins on page 2 and 7 of our review.

2. “The authors should elaborate more on the role of actin and myosin in autophagy-related diseases.”
A paragraph summarising the pathologies arising from mutations in NMM2A, MYO6 and MYO1C and their potential link to autophagy-related disease has been included at the end of the summary on page 11.

Referee 2
General comment:
1. “the authors would better integrate the autophagy part (the first 4 pages) with the actin/myosin part.”
The mauscript has been rearranged accordingly.

2. “…the authors should aim to include more details about autophagy”

Extra details whether myosin/actin play a role in selective or starvation-induced autophagy have now been included. However, we feel it is not appropriate to mention too much experimental detail on autophagy induction/inhibition, since this will confuse the general myosin/actin audience of this review.

3. “This is sometimes problematic as it can be misleading -- e.g. on page 6 when they discuss ref 65 (Lee et al; cortactin-HDAC6) they do not mention that this study found that HDAC6 and F-actin assembly were completely dispensable for starvation-induced autophagy.”
We have added the finding that F-actin was dispensable for starvation-induced autophagy and have clarified that their findings relate to selective autophagy as follows: “The actin cytoskeleton is also required for the fusion of autophagosomes with lysosomes (stage 3, Figure 1) during selective autophagy, but is dispensable for starvation-induced autophagy.”

4. “The authors should also aim to better describe any controversial findings, e.g. in ref 54 (Aguilera et al) it was found that actin functions at a very early stage of autophagosome formation, before association of LC3, whereas Mi et al (ref 57) LC3 is seen together with the actin puncta.”

After reviewing the overall literature on actin and myosin in autophagy, we feel that there is ample evidence that the actin cytoskeleton is required at several steps along the pathway, during autophagosome biogenesis and elongation. Therefore, the data presented in the two publications mentioned by referee 2 are most likely not to be controversial, but may simply be due to different probes, cell systems and inhibitors or modulators.
Nevertheless, to clarify that Aguilera et al found actin at the early autophagosome formation stage before association with LC3, we have added “before the association of LC3” to the text to highlight this: “Actin filaments have been shown to colocalize with early omegasomes before the association of LC3 suggesting a role for actin in the initial stages of autophagosome formation linked to the PI3P generation step.”

5. “By activating NMM2A, the ULK complex regulates the delivery of ATG9-containing vesicles from the TGN to the expanding phagophore (stage 2, Figure 1), thereby providing the membrane essential for biogenesis (86).”

This sentence has now been modified and reads as follows: “ The ULK complex has been shown to regulate the delivery of ATG9-containing vesicles from the TGN to the expanding phagophore (stage 1, Figure 1), thereby providing the membrane essential for biogenesis.”


Minor comments:

1. “Page 2: add a couple references to the Autophagy introduction.”
We have added the following references to the autophagy introduction: Kistakis and Tooze, Trends in Cell Biology, 2016 and Kaur and Debnath, Nat Rev Mol Cell Biol, 2015.

2. “Page 3, stage 2: they should add references to endosomes as sources of autophagy membrane (Longatti et al, JCB 2012; Knævelsrud et al, JCB 2013; Puri et al, Cell 2013).”

These references have been added.

3. “Page 3, stage 3: when citing LC3 conjugation to PE they should include the original paper; Kabeya et al, EMBO J, 2000 (ref 16 here) in addition to refs 14-15. It is not shown in ref 16 that LC3 is required for autophagosome closure and this is also highly debated in the field. They should also include a reference to amphisomes.”

We have added the Kabeya et al., EMBO J, 2000 reference to the LC3 conjugation to PE sentence and removed the statement that LC3 is required for autophagosome closure so that the sentence now reads: “LC3 is required for the elongation and remains associated with the completed autophagosome, while omegasomes are retracted back into the ER.” We have also added two references to amphisomes: Berg et al., J Biol Chem, 1998 and Tooze et al., J Cell Biol, 1990.

4. “Page 3, stage 4: they should also mention that degradation products can be recycled to the cytosol and reused.”

We have modified the sentence so that it mentions that degradation products can be recycled and it now reads: “In this acidic environment, lysosomal hydrolases degrade the contents and recycle these metabolites, including amino acids, fatty acids, glucose, and iron back to the cytosol.”

5. “The authors should be consistent in their citations, sometimes they write “as recently reviewed …” when they cite reviews, other times they cite a review without mentioning that this is a review.”

We have removed the “…as recently reviewed” statements to ensure consistency.

6. “Page 3; they should mention that not all forms of selective autophagy are ubiquitin-dependent.”

We have added the Khaminets et al., Trends in Cell Biology, 2016 reference and have included ubiquitin-independent means in the sentence: “They recognise and capture their cargoes marked for degradation with a ubiquitin tag via their ubiquitin-binding domains (UBDs) or via ubiquitin-independent means and simultaneously bind to LC3 on autophagosomal membranes via their LC3 interacting region (LIR) motif (Figure 1).”

7. “Page 4, second chapter; “In contrast, TAX1BP1 shows no preference and binds equally to LC3B and LC3C (22), which allows recruitment of a wider variety of membranes containing different LC3 isoforms and may suggest less specialised roles of TAX1BP1 in bacterial autophagy.” We do not know if the different LC3 (and GABARAP) isoforms are found in different membranes, so it is difficult to conclude about specificity.”

We have removed the section on LC3 (and GABARAP) isoforms as we do not go into further details on LC3 isoforms in subsequence sections of the review.

8. “Page 4: “…require TBK1-dependent enhancement of their ability to bind poly-ubiquitin,…” This should be explained better.”

We have changed the sentence and included a new paper (Richter et al., PNAS, 2016) so that it is clear that TBK1 phosphorylates the autophagy receptors thereby enhancing their ability to bind ubiquitin chains as follows: “OPTN and NDP52 are the primary (and to a lesser extent TAX1BP1) yet functionally redundant autophagy receptors that require Tank-binding kinase 1 (TBK1)-dependent phosphorylation to enhance their ability to bind ubiquitin chains, whereas NBR1 and p62 are dispensable for mitophagy.”

9. “Page 6, first paragraph: the authors write “Additional myosin motors may be required to actually translocate the membrane vesicles along actin filaments, as activated NMM2A, when assembled into myosin minifilaments, is unlikely to function as a cargo transporter.” Why not?”

We have clarified this as follows: “Additional myosin motors may be required to actually translocate the membrane vesicles along actin filaments, as activated NMM2A, when assembled into myosin mini-filaments, is unable to bind cargo via its C-terminal tail domain and thus function as a transporter.”

10. “Page 8, right panel: The authors should use be consistent in their use of the term autophagy receptor. Sometimes they use adaptor proteins instead, e.g. “MYO6 has been suggested to undergo monomer-dimer transitions regulated by cargo adaptor proteins (95).” This mix-up is confusing in the figure, where a red bar is called autophagy receptor in the key inset, while adaptor is used in the figure.”

We have gone through the manuscript and when we use “cargo adaptor proteins”, we consistently are referring to MYO6’s binding partners that also include the autophagy receptors. However, when we use “autophagy receptors,” we are only referring to the autophagy receptors. We thank the reviewer for noticing the inconsistency in Figure 1 and have adjusted it so that it is clear that the pink rectangle refers to the autophagy receptors only.

11. “Page 9, first paragraph: “Thus, OPTN, NDP52 and TAX1BP1 may have multiple roles in autophagy: initially acting as receptors for cargo recognition and autophagosome biogenesis and then in later stages, by binding directly to MYO6, they may be involved in autophagosome maturation and fusion with the lysosome.” MYO6 could have autophagy-receptor independent functions in autophagosome maturation!”

We have added a sentence to include the possibility that MYO6 has autophagy receptor-independent functions in autophagosome maturation: “However, it should be stressed that given MYO6’s involvement in diverse membrane trafficking pathways, it could also have autophagy receptor-independent functions in autophagosome maturation.”

12. “The figure: is nice, but maybe a bit crowded. The box showing actin within the omegasomes should be moved to under stage 1: initiation.”

We have moved the “omegasome” box underneath “stage 1a: initiation” as requested.

13. “The following references are not complete in the reference list and should be checked: 22,26, 32, 33, 46, 60, 68.”

References 22, 26, and 32 were already complete. We thank the reviewer for noticing that the references 33, 46, 60, 68 were incomplete and we have updated all of these references.

14. “Ref. 64; the original paper should be cited (Kast et al, Curr Biol 2015) instead of the Autophagy puncta.”

We have cited the Kast et al., Curr Biol, 2015 paper instead of the autophagy puncta in this place.

We would like to thank you and the two reviewers for taking the time to critically evaluate our manuscript and for their very helpful comments and suggestions. We feel that after taking their suggestions on board our manuscript has been very much improved.

Best wishes,

Folma Buss
